# Early prediction of postoperative liver dysfunction and clinical outcome using antithrombin III-activity

**DOI:** 10.1371/journal.pone.0175359

**Published:** 2017-04-13

**Authors:** David Pereyra, Florian Offensperger, Florian Klinglmueller, Stefanie Haegele, Lukas Oehlberger, Thomas Gruenberger, Christine Brostjan, Patrick Starlinger

**Affiliations:** 1 Department of Surgery, Medical University of Vienna, General Hospital, Vienna, Austria; 2 Core Unit for Medical Statistics and Informatics, Medical University of Vienna, Vienna, Austria; 3 Department of Surgery I, Rudolfstiftung Hospital, Vienna, Austria; Istituto Mediterraneo per i Trapianti e Terapie ad Alta Specializzazione, ITALY

## Abstract

**Background and aims:**

Antithrombin III (ATIII) has been reported to be associated with liver pathologies and was shown to predict outcome in patients undergoing liver resection for hepatocellular carcinoma. We now aimed to assess whether perioperative ATIII-activity could predict postoperative outcome in patients without underlying liver disease, as well as in a routine clinical setting of patients undergoing hepatic resection.

**Methods:**

ATIII-activity was evaluated preoperatively and on the first (POD1) and fifth day after liver resection in a retrospective evaluation cohort of 228 colorectal cancer patients with liver metastasis (mCRC). We further aimed to prospectively validate our results in a set of 177 consecutive patients undergoing hepatic resection.

**Results:**

Patients developing postoperative liver dysfunction (LD) had a more pronounced postoperative decrease in ATIII-activity (P<0.001). ATIII-activity on POD1 significantly predicted postoperative LD (P<0.001, AUC = 84.4%) and remained independent upon multivariable analysis. A cut-off value of 61.5% ATIII-activity was determined using ROC analysis. This cut-off was vital to identify high-risk patients for postoperative LD, morbidity, severe morbidity and mortality (P<0.001, respectively) with a highly accurate negative predictive value of 97%, which could be confirmed for LD (P<0.001) and mortality (P = 0.014) in our independent validation cohort. Further, mCRC patients below our cut-off suffered from a significantly decreased overall survival (OS) at 1 and 3 years after surgery (P = 0.011, P = 0.025).

**Conclusions:**

The routine laboratory parameter ATIII-activity on POD1 independently predicted postoperative LD and was associated with clinical outcome. Patients with a postoperative ATIII-activity <61.5% might benefit from close monitoring and timely initiation of supportive therapy.

**Trial registration:**

ClinicalTrials.gov NCT01700231

## Introduction

In a great number of patients suffering from liver malignancies surgical resection represents the treatment of choice and is frequently the only potentially curative treatment option[1–4]. It is important to preserve at least 20–25% of healthy liver volume, while resecting every radiological and macroscopically detectable tumour, to ensure sufficient postoperative liver function[5]. However, postoperative morbidity may compromise patients’ outcome and can often be related to a delay in liver regeneration[6, 7].

As a member of the serpin family Antithrombin III (ATIII) is a main inhibitor of proteinases active in coagulation such as thrombin and factor Xa[8–10]. ATIII is primarily synthesized by hepatocytes[11]. Furthermore, levels of ATIII have been shown to be altered in patients with liver disease[12, 13]. Recently, ATIII was reported to be a valuable marker for clinical outcome in hepatocellular carcinoma (HCC) patients undergoing curative intended liver resection[14–16]. In particular, ATIII has been proposed as a marker for overall and progression free survival in HCC patients[14]. Moreover, Mizugichi et al. showed a predictive potential of ATIII for postoperative liver dysfunction (LD) in a cohort of 158 HCC patients[15]. It is well known, that HCC is often associated with cirrhosis[17] or fatty liver disease[18]. However, the value of ATIII as a predictive marker in colorectal cancer liver metastases (mCRC) patients undergoing liver resection, who commonly do not suffer of primary liver pathologies[19], has not been investigated so far. Of note, while systemic levels of other factors involved in the coagulation cascade are largely affected by systemic treatments, postoperative substitution, deficiencies and diseases, ATIII seems to be comparably less affected and seems to be primarily altered by mutations, consumption or hepatocellular dysfunction[20, 21].

Within this study, we now aimed to explore the role of perioperative dynamics of ATIII as a predictive marker in a retrospective cohort of our prospectively maintained institutional database including a homogenous cohort of mCRC patients undergoing liver resection. Importantly, we aimed to further validate our results in a prospective validation cohort, consecutively including patients undergoing liver resection within a routine clinical setting.

## Patients and methods

### Study cohorts

Within our retrospective evaluation cohort, patients suffering from mCRC, resected between March 2001 and December 2009, were included using our prospectively maintained institutional database. To confirm our findings in a routine clinical setting a prospective validation cohort was recruited from January 2010 to October 2014. The extent of resection was characterized following the IHPBA Brisbane 2000 nomenclature in minor (< 3 liver segments) and major (≥ 3 liver segments)[22]. Blood samples were collected routinely prior to surgery (PRE-OP) as well as on the first (POD1) and fifth (POD5) day after liver resection. The study was approved by the institutional ethics committee ((#424/2010; #2032/2013) All patients gave written informed consent. Furthermore the study has been registered at a clinical trials registry (ClinicalTrials.gov Identifier: NCT01700231).

### Definition and classification of postoperative LD and morbidity

Postoperative LD was classified according to the ISGLS criteria[23]. Briefly, the criteria were met if serum bilirubin (SB) concentration and prothrombin time (PT) did not reach normal levels on POD5 after liver resection or thereafter. If SB and PT differed from standard values prior to the operation the criteria were fulfilled when both parameters were elevated for two succeeding days on or after POD5. Additionally, patients who reached normal SB or PT values before POD5 and thus had no further routine blood collection were considered as “no LD”.

Morbidity after surgery was assessed using the classification described by Dindo et al.[24]. The severity of postoperative complications was sub-classified in grade I-V. In case of multiple complications per patient, the most serious one was used for analysis. Further, patients with morbidity grade III-V were classified as patients with severe morbidity (SM).

Postoperative mortality was defined as death within 90 days after surgery.[25]

### Quantification of blood parameters

All perioperative parameters of liver function (SB, PT, aspartate aminotransferase (AST), alanine aminotransferase (ALT), gamma-glutamyl transferase (GGT), alkaline phosphatase (AP), and albumin) as well as C-reactive protein (CRP), fibrinogen and platelet counts were measured in appropriate samples by routine laboratory blood tests. Moreover, ATIII-activity was assessed in routine laboratory blood tests and used as a surrogate parameter for absolute levels of ATIII.

### Statistical analysis

All analyses are performed using data from subjects with valid marker values only. Statistical analyses were carried out using SPSS software (SPSS, Inc., Chicago, IL, USA, Version 23) and GNU R version 3.2.3.

#### Exploratory analysis of the evaluation samples

We compared ATIII-activity distributions between subjects with and subjects without LD, morbidity, SM and mortality using boxplots as well as Wilcoxon-Mann-Whitney U-Tests. Boxplot illustrations are given without outliers and extreme values to improve resolution of the interquartile ranges. Given the exploratory character of this analysis corresponding p-values are reported unadjusted. Furthermore, we fit receiver operating characteristic (ROC) curves for postoperative ATIII-activity for the prognosis of LD and mortality within 90 days after surgery. To further elucidate clinical relevance of ATIII-activity in the prediction of liver dysfunction following partial hepatectomy, we aimed to compare it to postoperative parameters that are already in clinical usage[26]. Therefore we performed a ROC analysis for postoperative ATIII-activity, fibrinogen, CRP, PT and SB. Furthermore, we included postoperative markers of liver injury, such as GGT, AST and ALT

To define a suitable cut-off value for the classification of patients with high risk for postoperative LD we computed the threshold value maximising the Youden-Index (i.e. the sum of sensitivity and specificity). We estimated corresponding values for sensitivity and specificity and computed bootstrap 95% confidence intervals. Proportions of subjects with LD, morbidity, SM and 90 day mortality are compared using relative proportions and the chi-square test (p-values are reported unadjusted).

#### Validation of the predictive potential of ATIII-activity

Prognostic potential of ATIII-activity on POD1 for the prognosis of LD, morbidity, SM and 90 day Mortality was validated using only data from the validation sample. We estimate sensitivity and specificity of ATIII-activity on POD1 using the cut-off chosen based on the evaluation sample. We tested whether subjects with high and low ATIII-activity (according to the cut-off chosen in the evaluation sample) show significantly different incidence of LD, morbidity, SM and postoperative mortality using chi-square tests. Considering this as the primary confirmatory analysis of this article, p-values of the four hypothesis tests are adjusted for multiplicity using the Bonferoni-Holm procedure. Test results are deemed statistically significant if the adjusted two-sided p-value is below 0.05.

#### Uni- and multivariable analysis

To further elucidate the prognostic value of ATIII-activity in combination with other potential markers we fit univariable and multivariable logistic regression models and perform a model selection using baseline variables and all available POD1 markers as candidate variables. Given the small number of LD cases in the validation sample, we combined the evaluation and validation sample for this analysis to achieve a better model fit. In a first step, separate univariable logistic regression models were performed for baseline characteristics and postoperative blood parameters. Second, we fit a multiple logistic regression model with stepwise forward selection to assess the effect of the respective parameters on LD status and postoperative mortality. The initial model included all parameters with a p-value below 0.05 in the univariable analysis. The p-value limit for a parameter to stay in the model was 0.1.

#### Classification tree

Finally to investigate potential interactions and nonlinear relationships between predictive markers and outcome variables we fitted binary classification tree [27] for the prediction of postoperative LD using postoperative markers and baseline characteristics. Tree depth was chosen to minimise the relative cross-validation error. Similar to the multivariable model we used the combined sample for this analysis due to the small number of cases with LD.

## Results

### Patients and cohorts

In total 405 patients were included into this study. The evaluation cohort consisted of 228 mCRC patients from our prospectively maintained institutional database and was analysed retrospectively. Succeeding, a prospective validation cohort consisting of 177 patients was recruited to confirm the results obtained within our evaluation set. The prospective validation cohort set out to reflect a routine clinical setting and accordingly consisted of mCRC (N = 84), HCC (N = 33), cholangiocellular carcinoma (CCC, N = 26), benign neoplastic entities (N = 16) and other liver pathologies requiring resection (N = 18). Baseline characteristics of both cohorts are given in Table 1. Median follow up for the evaluation and validation cohort were 67 and 38 months, respectively.

**Table 1:** Patients demographics.

Parameter	Entire collective Median (range) N (%)	Evaluation cohort Median (range) N (%)	Validation cohort Median (range) N (%)
**Age**	63 (22–89)	62 (28–83)	63 (22–89)
**Sex**			
Male	253 (62.5%)	143 (62.7%)	110 (62.1%)
Female	152 (37.5%)	85 (37.3%)	67 (37.9%)
**Neoplastic entity**			
mCRC	312 (77.0%)	228 (100.0%)	84 (47.5%)
HCC	33 (8.1%)	0 (0.0%)	33 (18.6%)
CCC	26 (6.4%)	0 (0.0%)	26 (14.7%)
Benign neoplasia	16 (4.0%)	0 (0.0%)	16 (9.0%)
Others	18 (4.4%)	0 (0.0%)	18 (10.2%)
**Hepatic resection**			
Minor	224 (55.3%)	151 (66.2%)	73 (41.2%)
Major	181 (44.7%)	77 (33.8%)	104 (58.8%)
**Preoperative Parameters**			
SB in mg/dl	0.62 (0.19–6.64)	0.63 (0.24–2.26)	0.62 (0.19–6.64)
PT in %	105 (40–150)	107 (45–147)	102 (40–150)
AP in U/l	99 (14–1111)	107.5 (42–1111)	86.5 (14–946)
AST in U/l	28 (5–496)	28 (5–496)	28.5 (15–404)
ALT in U/l	24 (2–410)	22 (2–410)	26.5 (6–346)
GGT in U/l	51 (9–968)	47 (9–968)	57.5 (11–699)
Albumin in g/l	41.4 (21.0–52.0)	41.0 (21.0–50.0)	42.0 (32.0–52.0)
Platelets in G/l	176 (49–503)	139 (49–503)	226.5 (86–492)
**Co-factors**			
CTx neoadjuvant	284 (70.1%)	210 (92.1%)	74 (41.8%)
Pringle maneuver	72 (17.8%)	23 (10.1%)	49 (27.7%)
RBC intraoperative	53 (13.1%)	40 (17.5%)	13 (7.3%)
**Morbidity**			
No morbidity	246 (60.7%)	150 (64.8%)	96 (54.2%)
I	32 (7.9%)	14 (6.1%)	18 (10.2%)
II	65 (16.0%)	43 (18.9%)	22 (12.4%)
IIIa	19 (4.7%)	6 (2.6%)	13 (7.3%)
IIIb	27 (6.7%)	9 (3.9%)	18 (10.2%)
IVa	10 (2.5%)	4 (1.8%)	6 (3.4%)
IVb	1 (0.2%)	0 (0.0%)	1 (0.6%)
V	5 (1.2%)	2 (0.9%)	3 (1.7%)
**Liver dysfunction**			
No LD	362 (89.4%)	202 (88.6%)	160 (90.4%)
ISGLS A	22 (5.4%)	15 (6.6%)	6 (3.4%)
ISGLS B	10 (2.5%)	5 (2.2%)	5 (2.8%)
ISGLS C	11 (2.7%)	5 (2.2%)	6 (3.4%)
**Postoperative follow up**			
Days at ICU	1 (0–42)	1 (0–42)	1 (0–26)
POH in days	8 (3–117)	9 (4–77)	8 (3–117)
90 days mortality	10 (2.5%)	7 (3.1%)	3 (1.7%)

mCRC = metastasized colorectal cancer, HCC = hepatocellular carcinoma, CCC = cholangiocellular carcinoma, SB = serum bilirubin, PT = prothrombin time, AP = alkaline phosphatase, AST = aspartate aminotransferase, ALT = alanine aminotransferase, GGT = gamma-glutamyltransferase, CTx = chemotherapy, RBC = red blood cells, LD = liver dysfunction, ISGLS = International Study Group for Liver Surgery, ICU = intensive care unit, POH = postoperative hospitalisation.

Missing data is visualized in S1 Table. Values for ATIII-activity could be obtained from the majority of patients prior to the operation as well as on POD1. Due to early postoperative dismissal and immediate postoperative death missing vales increase on POD5 (53% in evaluation set, 49% in validation set). Postoperative follow up concerning the outcome parameters LD (patients dismissed prior to POD5 with already normal PT and SB were classified as noLD–see methods section), morbidity, postoperative mortality, as well as OS, could be recorded from every patient.

### Postoperative levels of ATIII-activity decrease after liver resection and correlate with extent of resection

**Fig 1:**
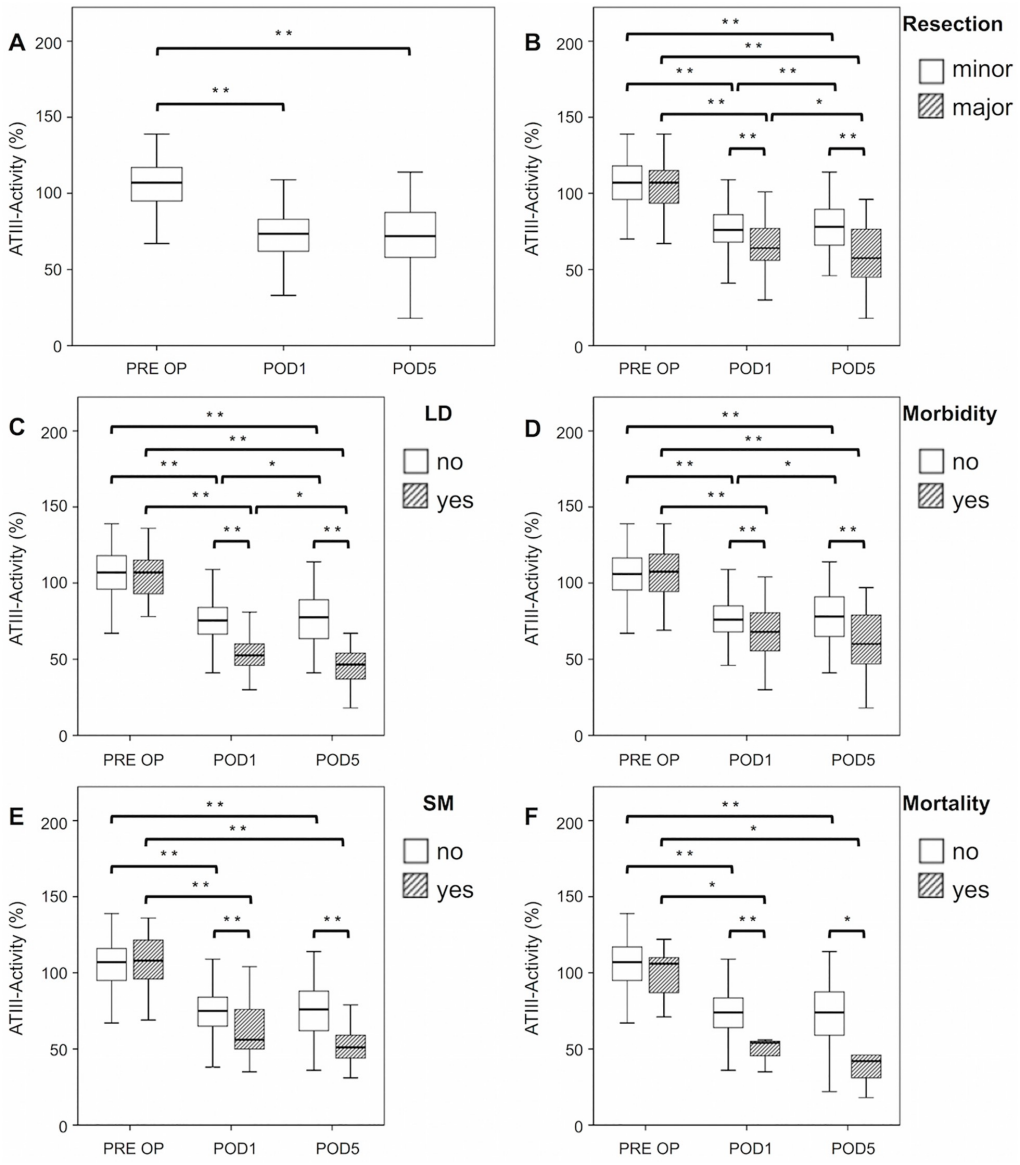
Perioperative ATIII-activity dynamics. ATIII-activity was evaluated prior to surgery (PRE OP), as well as on the first (POD1) and fifth (POD5) postoperative day. Perioperative dynamics are shown in A for all patients of the evaluation cohort. Patients were divided in groups according to their resection extent (B). Further, dynamics of ATIII-activity in patients with and without postoperative liver dysfunction (LD) were assessed and are illustrated in C. Moreover, patients were grouped depending on postoperative outcome. ATIII-activity is shown for patients with or without postoperative morbidity (D), postoperative severe morbidity (SM) (E) and mortality within 90 days after surgery (F). * P < 0.05; ** P < 0.005.

First, we investigated the perioperative dynamics of ATIII-activity after liver surgery in our evaluation cohort (Fig 1A). We observed a significant decrease in ATIII-activity on POD1 (median ATIII PRE OP = 107%, median ATIII POD1 = 73.5%, P<0.001), which persisted upon POD5 (median ATIII POD1 = 73.5%, median ATIII POD5 = 72%, P = 0.347). Moreover, we found that ATIII-activity followed the extent of hepatic resection (Fig 1B). While no preoperative difference in ATIII-activity was observed (median ATIII before minor resection = 107%, median ATIII before major resection = 107%, P = 0.634), we found that patients undergoing major resection had a more pronounced decrease in ATIII-activity compared to patients undergoing minor resection on POD1 (median ATIII after minor resection = 76%, median ATIII after major resection = 64%, P<0.001) as well as on POD5 (median ATIII after minor resection = 78%, median ATIII after major resection = 57.5%, P<0.001). Interestingly, patients receiving minor resections were able to partially restore levels of ATIII-activity from POD1 to POD5 (P = 0.004), while patients with major resection further decreased significantly (P = 0.047).

### Postoperative ATIII-activity is associated with LD after liver resection

We further aimed to evaluate ATIII-activity in patients with and without postoperative LD (Fig 1C). In our evaluation cohort a total of 26 patients suffered from LD after surgery. Preoperative ATIII-activity did not differ between both groups (median ATIII no LD = 107%, median ATIII LD = 107%, P = 0.517), whereas patients developing LD after surgery had extensively reduced levels of ATIII on POD1 (median ATIII no LD = 75.5%, median ATIII LD = 52.5%, P<0.001) and POD5 (median ATIII no LD = 77.5%, median ATIII LD = 46.5%, P<0.001). Of note, regarding the postoperative time course, ATIII-activity increased in patients who did not suffer from LD (P = 0.018), whereas it decreased significantly in patients who developed postoperative LD (P = 0.009).

### Postoperative ATIII-activity correlates with poor clinical outcome and predicts mortality within 90 days

We further evaluated ATIII-activity concerning postoperative morbidity according to Dindo et al.[24] (Fig 1D). In total 81 patients in the evaluation cohort suffered from postoperative morbidity (grad I-V according to Dindo et al.[24]) during their postoperative course. These patients were found to have significantly lower levels of ATIII-activity on POD1 (median ATIII no morbidity = 76%, median ATIII morbidity = 68%, P = 0.001) and on POD5 (median ATIII no morbidity = 78%, median ATIII morbidity = 60%, P<0.001), without showing significant differences in their preoperative ATIII-activity (median ATIII no morbidity = 106%, median ATIII morbidity = 107.5%, P = 0.766). Patients without postoperative morbidity were able to partially restore ATIII (P = 0.023), while patients with postoperative morbidity did not recover but tended to further decrease (P = 0.238) during the postoperative period.

We further sub-classified postoperative complications in severe morbidity, namely patients developing postoperative morbidity grade III-V according to Dindo et al.[24] (Fig 1E; N = 25). As for morbidity, patients developing severe morbidity had significantly decreased levels of ATIII-activity on POD1 (median ATIII no severe morbidity = 75%, median ATIII severe morbidity = 56%, P<0.001) and on POD5 (median ATIII no severe morbidity = 76%, median ATIII severe morbidity = 51%, P = 0.001). Of note, preoperative ATIII-activity in patients suffering from severe morbidity in the postoperative time course did not differ from patients who recovered well after surgery (median ATIII no severe morbidity = 107%, median ATIII severe morbidity = 108%, P = 0.732).

Seven patients died within 90 days after surgery. There were no preoperative differences in ATIII-activity levels (P = 0.459, Fig 1F). However, patients that suffered from postoperative mortality had substantially lower ATIII-activity on POD 1 (median ATIII no mortality = 74%, median ATIII mortality = 54%, P<0.001), as well as on POD5 (median ATIII no mortality = 74%, median ATIII mortality = 42%, P = 0.025).

### Postoperative ATIII-activity challenges established postoperative markers of LD

**Fig 2:**
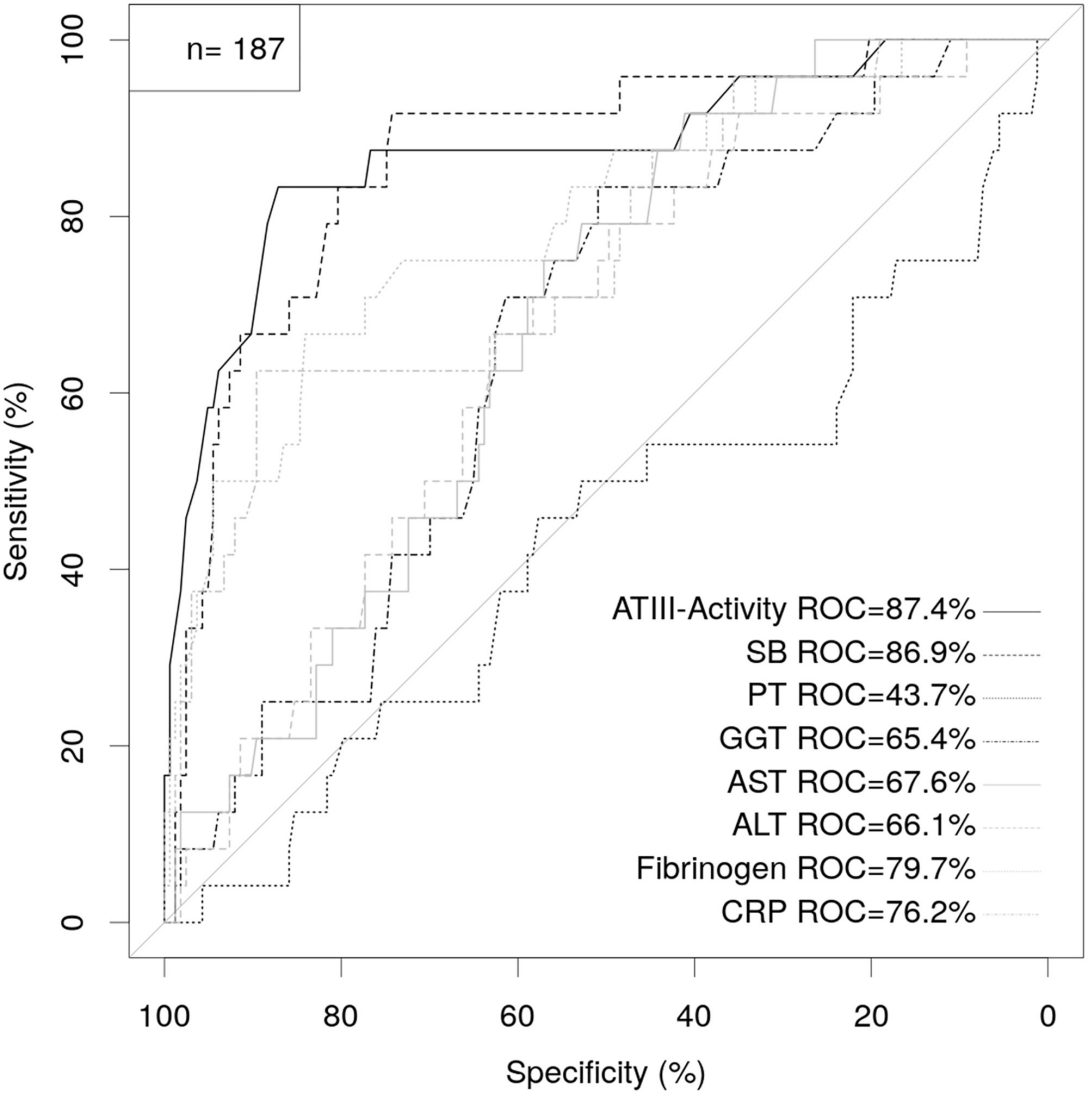
ATIII-activity on POD1 competes other markers in the prediction of postoperative LD. Predictive value of ATIII-activity, CRP, fibrinogen, SB, PT, AST, ALT and GGT on POD1 to predict postoperative LD using receiver operating characteristics (ROC) analysis was compared.

Next, we performed a ROC analysis for postoperative ATIII-activity, fibrinogen, CRP, PT, SB, as well as GGT, AST and ALT in a subgroup of patients that do not miss any of those markers (N = 187). Strikingly, our results showed that ATIII-activity on POD1 was the strongest predictor of postoperative LD in our study cohort (AUC = 87.4%, P<0.001, 95%-CI: 0.783–0.964, Fig 2).

### Values of ATIII-activity on POD1 below 61.5% specifically identify patients with postoperative LD and poor clinical outcome

**Fig 3:**
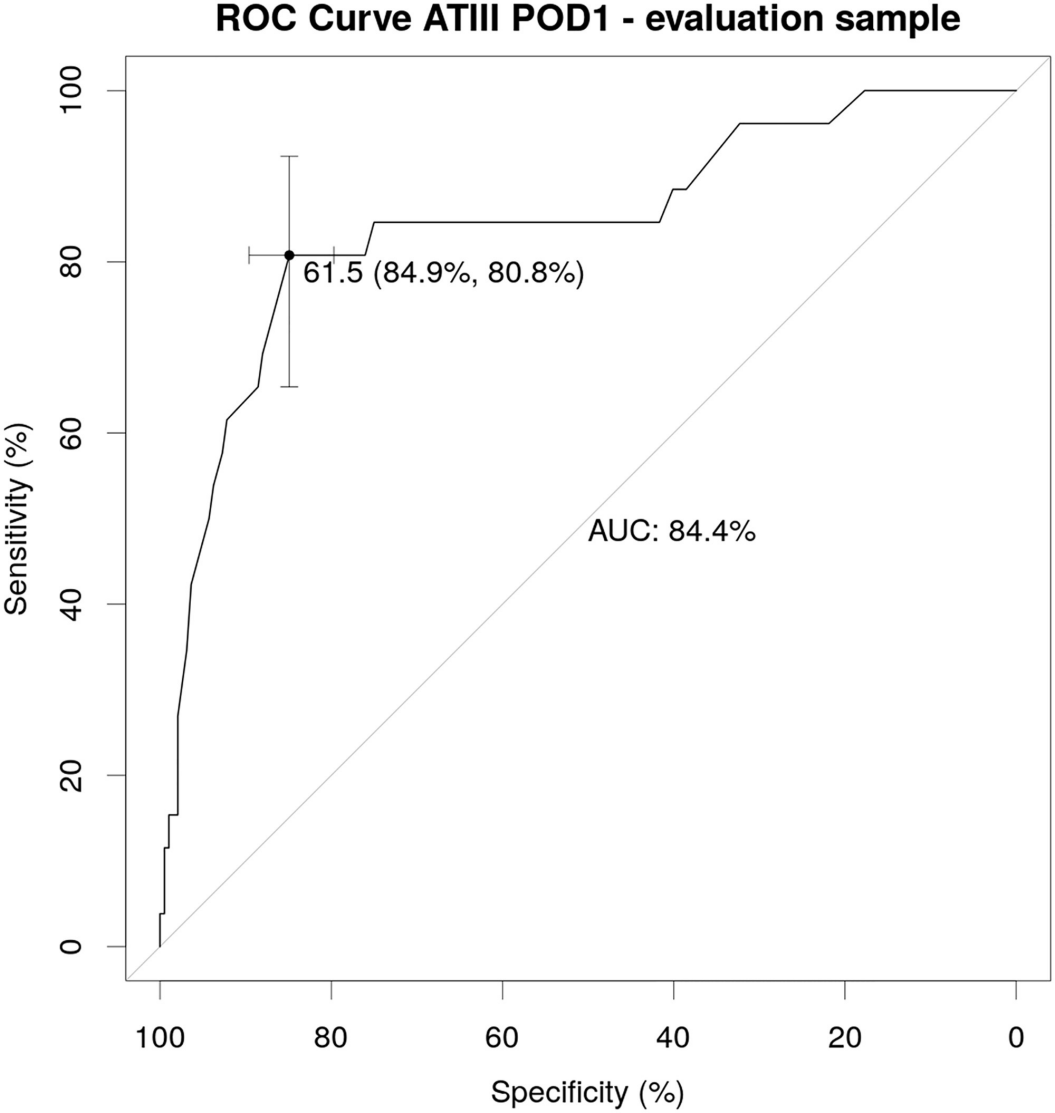
ATIII-activity on POD1 predicts postoperative LD. Receiver operating characteristic (ROC) curve for ATIII-activity on POD1 in the evaluation sample. Point with confidence bars gives sensitivity and specificity for the cut-off maximizing the sum of sensitivity and specificity.

To further assess the value of ATIII-activity to predict postoperative LD after liver resection we performed a ROC analysis in our evaluation cohort. Accordingly, we found that ATIII-activity on POD1 was able to significantly predict postoperative LD (AUC = 84.4%, P<0.001, 95%-CI: 0.753–0.936, Fig 3). To identify high-risk patients that will most likely develop postoperative LD, we defined a cut-off value at 61.5% ATIII-activity on POD1 with a high specificity of 84.9% (95%-CI: 0.797–0.896) and an equally good sensitivity of 80.8% (95%-CI: 0.654–0.962). This leads to a positive predictive value (PPV) of 42% and a negative predictive value (NPV) of 97%.

**Fig 4:**
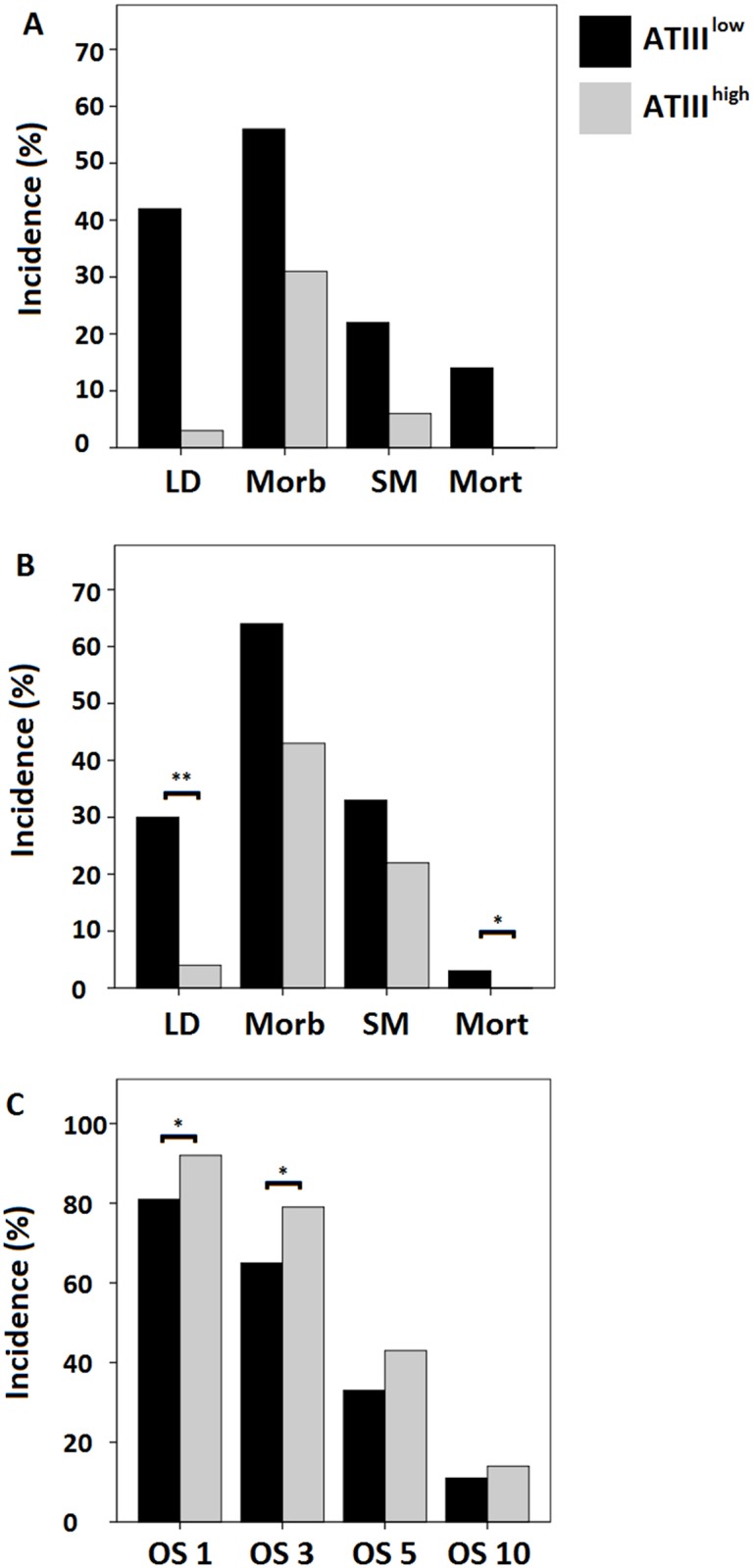
Patients with low postoperative ATIII-activity suffer from poor clinical outcome. Using a cut-off of 61.5% ATIII-activity, patients were divided in a high-risk group (ATIII^low^) and a low-risk group (ATIII^high^). Incidence of postoperative LD, morbidity, severe morbidity (SM) and mortality are illustrated according to risk groups in our evaluation cohort (A) and were confirmed in our validation cohort (B). Overall survival (OS in %) for mCRC patients in the entire collective was assessed for both risk groups after 1, 3, 5 and 10 years (C). * P < 0.05; ** P < 0.005.

According to this cut-off, a high-risk group (ATIII^low^) consisting of 50 patients was identified, while the remaining 168 patients were classified as a low-risk group (ATIII^high^). Patients within our high-risk group suffered more frequently from postoperative LD (P<0.001, 21 of 50 [42%] in ATIII^low^ vs. 5 of 168 [3%] in ATIII^high^) as shown in Fig 4A.

Furthermore, we aimed to evaluate if patients within our high-risk group suffered from an increased incidence of postoperative morbidity and mortality. Indeed, patients in our high-risk group developed postoperative morbidity (P = 0.001, 28 of 50 [56%] in ATIII^low^ vs. 52 of 168 [31%] in ATIII^high^), postoperative severe morbidity (P = 0.001, 11 of 50 [22%] in ATIII^low^ vs. 10 of 168 [6%] in ATIII^high^) and postoperative mortality (P<0.001, 7 of 50 [14%] in ATIII^low^ vs. 0 of 168 [0%] in ATIII^high^) with a significantly higher incidence. Results for our evaluation cohorts are visualized in Fig 4A.

### Prospective validation cohort confirms predictive potential of ATIII-activity and cut-off value

After having observed a predictive potential of postoperative ATIII-activity for postoperative LD and clinical outcome in our evaluation cohort of 228 patients, we aimed to validate our findings in a prospective validation cohort of 177 consecutive patients, reflecting a routine clinical setting. To validate the predictive potential of postoperative ATIII-activity for LD after liver resection, again ROC analysis was performed. Interestingly, results showed an equally good predictive potential for ATIII-activity with an AUC of 84.2% (P<0.001, 95%-CI: 0.752–0.932) and using 61.5% as a cut-off yields a specificity of 85% (95%-CI: 0.790–0.902) and a sensitivity of 62.5% (95%-CI: 0.375–0.813 95%). Moreover, comparison of ATIII-activity to established markers of postoperative LD as performed in the evaluation cohort via ROC analysis was performed and strikingly, even in the validation cohort ATIII-activity provided the highest AUC of 83.8% (P<0.001, 95%-CI: 0.741–0.935, S1 Fig).

Consequently, the validation cohort was divided in risk groups using the same cut-off value as determined in the evaluation cohort. In line with the findings in the evaluation cohort, patients in the validation cohort within the high-risk group had a significantly higher incidence of postoperative LD (P<0.001, 10 of 33 [30%] in ATIII^low^ vs. 6 of 136 [4%] in ATIII^high^) and postoperative mortality (P = 0.014, 3 of 30 [10%] in ATIII^low^ vs. 0 of 136 [0%] in ATIII^high^), as visualized in Fig 4B. Incidences for postoperative morbidity and severe morbidity did not significantly differ between risk groups in our validation cohort. However, a similar tendency as in the evaluation cohort could be observed for both morbidity (P = 0.096, 21 of 33 [64%] in ATIII^low^ vs. 58 of 136 [43%] in ATIII^high^) and severe morbidity (P = 0.175, 11 of 33 [33%] in ATIII^low^ vs. 30 of 136 [22%] in ATIII^high^). Of note, Bonferoni-Holm correction for multiple testing was applied as stated in the methods section.

### ATIII-activity remains an independent marker for postoperative LD and postoperative mortality upon multivariable analysis and is associated with overall survival

As we had observed a significant predictive potential of ATIII-activity for LD after hepatic resection, we aimed to explore if ATIII-activity on POD1 can independently predict postoperative LD. Therefore, multivariable analysis (MVA) was performed including the entire cohort of 405 patients (evaluation and validation cohort) to achieve sufficient statistical power. Extent of resection, ATIII-activity, SB, PT, GGT, AST, ALT, and albumin gave p-values below 0.05 in the univariable models for LD and were consequently included in the initial mulitivariable model. After step-wise forward selection extent of resection, ATIII, SB and ALT remained. The results from the final model fit are shown in Table 2A.

**Table 2:** Multivariable analysis for liver dysfunction and 90 days mortality.

**A. Multivariable analysis for liver dysfunction in the entire collective.**			
**Parameters**	**OR**	**95% CI**	**P-Value**
Extent of Resection	3.706	1.271–10.805	0.016
ATIII-Activity	0.889	0.853–0.926	<0.001
SB	1.508	1.218–1.867	<0.001
ALT	1.001	1.000–1.002	0.001
**B. Multivariable analysis for 90 days mortality in the entire collective.**			
**Parameters**	**OR**	**95% CI**	**P-Value**
ATIII-Activity	0.863	0.809–0.920	<0.001

OR = odds ratio, CI = confidential interval, ATIII = antithrombin III, SB = serum bilirubin, ALT = alanine aminotransferase

Further, we aimed to explore whether postoperative ATIII-activity can independently predict death within 90 days after liver resection. Again, we performed a MVA, including all significant parameters for postoperative mortality from univariate analysis, which were age, ATIII-activity, SB, PT, GGT, AST. Interestingly, after step-wise forward selection exclusively ATIII-activity remained highly significant with an odds ratio of 0.863 (95% Confidence interval 0.809–0.920, p-value <0.001; Table 2B).

Moreover, we aimed to evaluate the association between postoperative ATIII-activity and long term overall survival (OS). To allow for a homogenous cohort, exclusively patients treated for mCRC (N = 312) were included in OS analyses. Hence we found, that postoperative ATIII-activity was associated with long term overall survival. In particular, ATIII-activity was significantly reduced for patients that died within the first (median ATIII survivors = 74%, median ATIII dead = 62%, P = 0.002), third (median ATIII survivors = 75%, median ATIII dead = 68%, P = 0.001), fifth (median ATIII survivors = 76%, median ATIII dead = 71%, P = 0.005) and up to tenth year after surgery (median ATIII survivors = 80%, median ATIII dead = 72%, P = 0.009). Furthermore, in regards to our proposed cut off, the low-risk group had a significantly higher overall survival rate after one (P = 0.011, 51 of 63 [81%] in ATIII^low^ vs. 216 of 235 [92%] in ATIII^high^) and three years (P = 0.025, 41 of 63 [65%] in ATIII^low^ vs. 185 of 235 [79%] in ATIII^high^), as shown in Fig 4C.

### Combination of ATIII-activity with SB on POD1 determined using a classification tree represents a useful tool for risk stratification in our cohort

**Fig 5:**
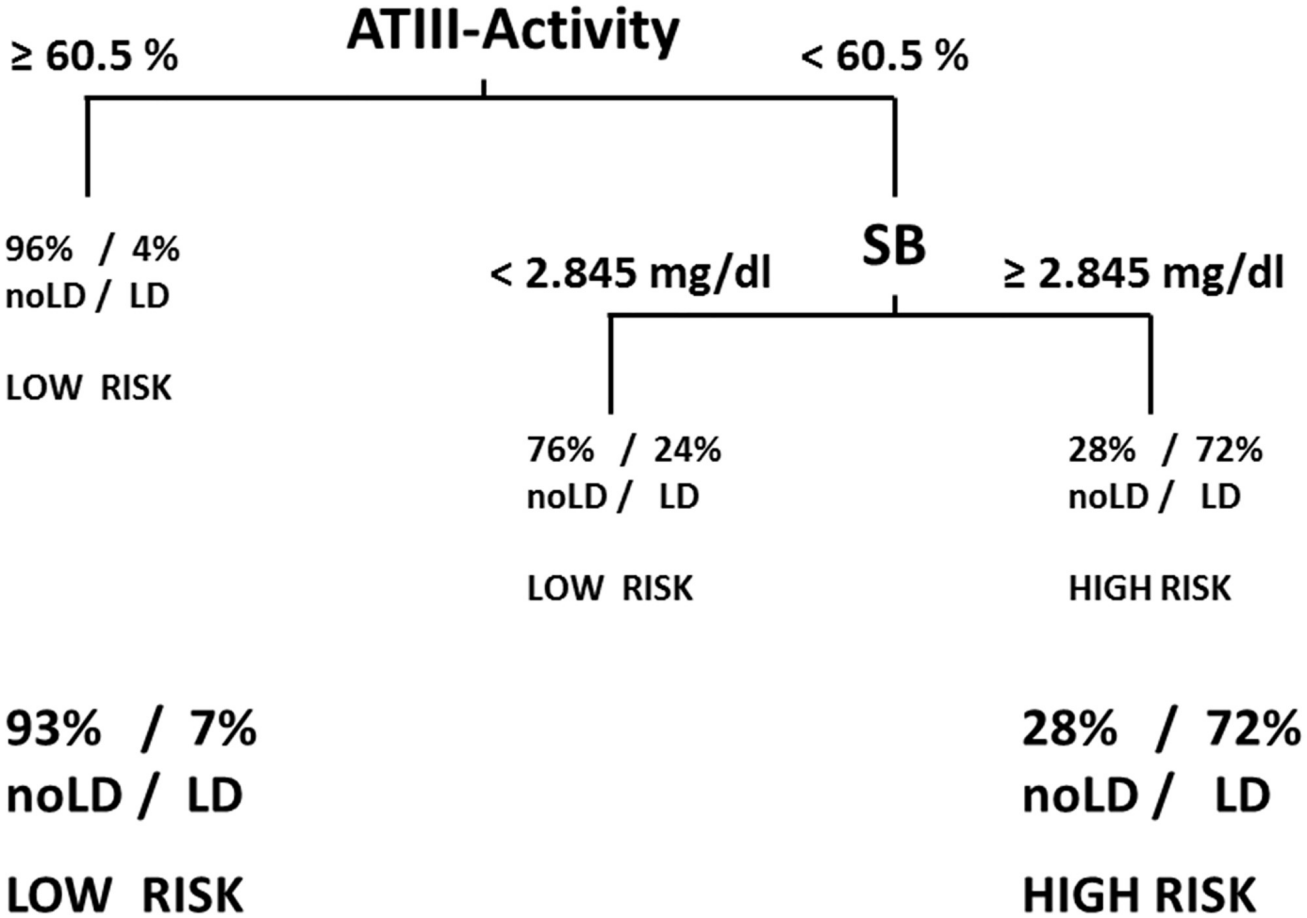
Decision tree for postoperative risk stratification. Combination of ATIII-activity and SB values on POD1 allows highly specific discrimination of patients risk for postoperative LD.

To investigate whether any interactions between postoperative markers or nonlinear relationships influence the predictive potential of ATIII-activity, we fited a binary classification tree to predict LD using all baseline characteristics and postoperative markers as candidate variables, identifying ATIII-acitivity as the variable with the best discriminatory capacity. After correction for overfitting the resulting classification tree first classified subjects with ATIII-activity levels above 60.5 (entire cohort) as low risk for LD and among the remaining subjects only those with SB values above 2.845 mg/dl as high risk patients. This provided a sensitivity of 42% and a specificity of 98% that corresponds to a PPV of 72% and an NPV of 93%. The resulting decision tree diagram is shown in Fig 5.

## Discussion

Despite substantial improvements in liver surgery, postoperative LD, morbidity and mortality still represent a major clinical concern[6, 7, 25]. In this study, we were able to demonstrate that low levels of ATIII-activity as early as on the first day after surgery could predict postoperative outcome in a retrospective evaluation cohort of 228 patients suffering from mCRC. To our knowledge, this represents the first clinical evidence for a close association of postoperative clinical outcome with ATIII-activity in patients without primary liver diseases. Importantly, we were further able to validate our results within a prospective validation set of 177 consecutive patients undergoing liver surgery in a routine clinical setting. Ultimately, we were able to document the independent association of ATIII-activity with postoperative LD and mortality upon multivariable analysis.

ATIII is an α_2_-globulin and mainly synthesized by the liver. Via inhibition of thrombin, factor Xa, but also of factors IXa, XIa, XIIa, tissue plasminogen activator (tPA), trypsin, plasmin, urokinase and kallikrein, ATIII acts as a physiologic anticoagulant[28]. It is well known that PT is prolonged by a series of conditions, including direct acting anticoagulants (among them argatroban, dabigatran, rivaroxaban, apixaban, edoxaban)[29] and intake of vitamin K antagonists[30], vitamin K deficiency[31], coagulation factor deficiency, lupus anticoagulans[32], polycitemia vera[33]. Similarly, it was shown that factor VII deficiency is frequent in patients with infections, neoplasia, trauma, nephrotic syndrome, left heart failure, penicillin intake and clearly in patients under vitamin K dependent anticoagulation[34, 35]. However, ATIII should only be altered due to mutations[36], consumption[37] or hepatocellular dysfunction[38, 39].

Our data support the hypothesis that ATIII—and subsequently ATIII-activity as a surrogate parameter for absolute amount of circulating ATIII—represents a synthesizing parameter of the liver as we observed a decrease after liver resection and a more pronounced reduction in patients undergoing major liver resection. As hemodynamic management after surgery is highly standardized at our institution and variability in postoperative fluid management is limited, we do not believe that hemodilution is responsible for the observed dynamics of ATIII-activity. Additionally, intraoperative blood loss is kept to a minimum. In fact, we could not observe significant differences in the distribution of postoperative ATIII-activity between patients that did or did not receive intraoperative blood transfusion (data not shown). Still, consumption of ATIII after liver resection is highly increased. This drastically shortens half-life of ATIII, which might suggest that hepatocellular synthesis may be the major determinant for postoperative levels of ATIII that are reflected by ATIII-activity values.

Besides its association with hepatocellular function, experimental studies suggest a potential pathophysiologic role of ATIII during liver regeneration. Preclinical data report on the regulatory effect of ATIII on intrahepatic inflammation and apoptosis[40]. In particular, ATIII has been shown to reduce liver apoptosis via induction of IGF-1 in a rodent model[41]. As IGF-I is known to be centrally involved during liver regeneration[42] ATIII might potentially act as an inducer of hepatic regeneration. In this context, we would like to point out that recently clinical data on the beneficial effect of postoperative ATIII administration was reported by Kuroda et al.[16]. Patients that received ATIII substitution after curative resection for HCC had a significantly lower risk of postoperative LD, suggesting a potential role of ATIII during liver regeneration. However, the authors hypothesized that ATIII might reduce incidences of LD through a suppression of coagulopathies[16]. Previously it was shown that ATIII reduces ischemia/reperfusion injury in a rodent model[40]. This might be due to reduced thrombosis. However, while thrombosis is a major effector in ischemia/reperfusion injury, its relevance in LD after partial hepatectomy remains poorly understood. In our entire cohort 7 patients developed coagulopathies (details given in Table 3). Yet, patients in our study cohort suffering from thromboembolic incidences did not significantly differ in terms of their ATIII-activity on POD1 (median ATIII no thromboembolisms = 73%, median ATIII thromboembolisms = 69%, P = 0.238). Further, we did not observe a correlation between thromboembolic occurrence and postoperative LD (r = 0.077, P = 0.120). This might suggest that additional effects of ATIII could support postoperative liver regeneration in patients without underlying liver disease. Still, due to the limited number of thromboembolic events, we are ultimately unable to sort out weather patients with low levels of ATIII-activity are prone to develop microthrombosis after liver resection.

**Table 3:** Detail on postoperative thromboembolic incidences.

Type of Thromboembolic Incidence	Patients (N = 7)
deep vein thrombosis	1
jugular vein thrombosis	3
postoperative portal vein thrombosis	2
pulmonary embolism	1

Besides possible therapeutic benefits of ATIII administration, the major finding of our investigation was the striking predictive potential of ATIII-activity for postoperative LD and clinical outcome in mCRC patients without underlying liver disease, as well as in a routine clinical setting of hepatic resections. Accordingly, ATIII-activity allowed to identify high-risk patients timely after liver resection. Interestingly, the severity of postoperative LD, scored from A to C following the ISGLS criteria[23] showed highly significant differences within our risk groups (S2A Fig). Further, incidence of grades of postoperative morbidity classified according to Dindo et al.[24] tended to be increased in our high-risk group (S2B Fig). Of note, only patients belonging to our high-risk group suffered from life threatening or lethal complications, graded with IVb and V according to Dindo et al.[24]. This striking association might allow us to tailor postoperative treatment for patients at risk shortly after liver surgery. Accordingly, growing evidence suggest that liver support devices such as the molecular adsorbent recirculating system (MARS) are primarily effective, if they are introduced early[43]. In this context ATIII-activity could be helpful to guide treatment decisions. Furthermore, high-risk patients might benefit from a timely radiologic assessment or precise monitoring of liver function parameters for early detection of postoperative LD. As patients suffering from postoperative LD are prone to develop infectious complications, decreased postoperative ATIII-activity levels could trigger rapid postoperative antibiotic therapy, if indicated.

Further, we want to point out that postoperative ATIII-activity was able to compete with established postoperative markers of LD. Importantly, ATIII-activity showed a superior potential to predict postoperative LD as compared to postoperative PT, which represents an internationally acknowledged marker for postoperative liver function. Of note, despite the fact that the ISGLS criteria use PT and SB on POD5 to define patients with postoperative LD, ATIII-activity showed a comparable predictive potential for postoperative LD on POD1 as SB in an independent manner as shown by MVA. These findings further underline the clinical importance of ATIII-activity in risk-assessment after liver resection. However, ultimately the combination of ATIII-activity with SB on POD1 within our classification tree model showed the highest predictive accuracy for postoperative LD and might represent the clinically most useful tool of our investigation (see also Fig 5). As the fitting algorithm identified the best cut-off for ATIII-activity in the pooled cohort (60.5%), the cut-off was close to, yet not exactly the same as our proposed cut-off of 61.5% identified in the homogenous evaluation cohort. Ultimately, an ideal cut-off for ATIII-activity will range between 60.5 to 61.5%. However, the fitted classification tree nicely illustrated, that after risk stratification via ATIII-activity, further subclassification using SB (cut-off of 2.845 mg/dl) may further improve predictive accuracy. Of note, ATIII-activity had highest discriminatory capacity in this model, which again points towards the use of this routine laboratory marker in patients undergoing liver resection.

An additional major finding of this study is the correlation of low levels of ATIII-activity and postoperative mortality. In our entire study collective 10 patients died in the postoperative period. Interestingly, death of all those patients could be predicted using our cut off for ATIII on POD1. In particular, 15.9% of our high-risk patients died within the first 90 postoperative days, whereas no postoperative mortality was observed in our low risk group (based on our entire collective). ROC analysis of ATIII-activity on POD1 showed a remarkable predictive potential with an AUC of 95.5% for postoperative 90 days mortality (P<0.001, 95%-CI: 0.916–0.994). Moreover, we showed that ATIII-activity on POD1 is significantly reduced in mCRC patients that died within 1, 3, 5 and 10 years after liver surgery. In line with these findings, again our ATIII-activity cut-off was able to predict survival within one and three postoperative years. Accordingly, this finding further underlines the predictive relevance of postoperative ATIII-activity for postoperative overall outcome.

Taken together, to our knowledge this study presents the first clinical evidence for a fundamental impact of ATIII-activity on postoperative risk stratification in a routine clinical setting of patients undergoing curative liver resection, while given clinical evidence primarily relays on HCC patients[14–16]. Postoperative ATIII-activity was found to be a valuable and independent predictor for postoperative LD and poor clinical outcome after hepatic resection. Additionally, preoperative ATIII-activity showed a predictive potential for postoperative mortality. Because ATIII-activity is easily assessable and included in most clinical routine laboratory reports, this parameter might reliably be used to identify high-risk patients timely after surgery that will benefit from close monitoring and early induction of supportive treatment. Although we implied ATIII as a synthesizing parameter of the liver, the protein might have an additional physiologic involvement during liver regeneration. However, this hypothesis needs further investigation. This is of specific clinical interest, as therapeutic efficiency of postoperative ATIII substitution might not be limited to HCC patients as reported previously[16], but be presumably beneficial in all patients undergoing liver resection independent of underlying liver disease.

## Supporting information

S1 Fig ATIII-activity on POD1 competes other markers in the prediction of postoperative LD in the validation cohort. Predictive value of ATIII-activity, CRP, fibrinogen, SB, PT, AST, ALT and GGT on POD1 to predict postoperative LD using receiver operating characteristics (ROC) analysis was compared.
(TIF)

S2 Fig ATIII-activity risk groups correlate with ISGLS grades for LD and Dindo et al. grades for morbidity. Patients were characterized in risk groups regarding their ATIII-activity levels, leading to a high-risk group (ATIII^low^) and a low-risk group (ATIII^high^). Incidences of LD graded following the ISGLS score system were compared for patients in our high- and low-risk group (A). (no LD: P<0.001, 52 of 83 [62%] ATIII^low^ vs 293 of 304 [96%] ATIII^high^; ISGLS-A: P<0.001, 17 on 83 [20%] ATIII^low^ vs 4 on 304 [1%] ATIII^high^; ISGLS-B: P = 0.003, 6 on 83 [7%] ATIII^low^ vs 4 on 304 [1%] ATIII^high^; ISGLS-C: P<0.001, 8 on 83 [10%] ATIII^low^ vs 3 on 304 [1%] ATIII^high^). Incidences for morbidity grades according to Dindo et al. were compared to our risk groups. (no Morbidity: P<0.001, 35 on 83 [42%] ATIII^low^ vs 196 of 304 [65%] ATIII^high^; Dindo I: P = 0.063, 11 on 83 [13%] ATIII^low^ vs 21 on 304 [7%] ATIII^high^; Dindo II: P = 0.565, 15 on 83 [18%] ATIII^low^ vs 47 on 304 [15%] ATIII^high^; Dindo IIIA: P = 0.270, 6 on 83 [7%] ATIII^low^ vs 13 on 304 [4%] ATIII^high^; Dindo IIIB: P = 0.557, 7 on 83 [8%] ATIII^low^ vs 20 on 304 [7%] ATIII^high^; Dindo IVA: P = 0.504, 3 on 83 [4%] ATIII^low^ vs 7 on 304 [2%] ATIII^high^; Dindo IVB: P = 0.055, 1 on 83 [1%] ATIII^low^ vs 0 on 304 [0%] ATIII^high^; Dindo V: P<0.001, 5 on 83 [6%] ATIII^low^ vs 0 on 304 [0%] ATIII^high^). * P < 0.05; ** P < 0.005.
(TIF)

S1 Table Information on missing data. For further detail refer to PONE-D-16-33767R1_FTC-SupportingInformation.docx.
(DOCX)
