# Supplementary figures and images for: Early prediction of postoperative liver dysfunction and clinical outcome using antithrombin III-activity
Source: PLoS One. 2017 Apr 13;12(4):e0175359. doi: 10.1371/journal.pone.0175359 (PMC5391027; doi:10.1371/journal.pone.0175359)

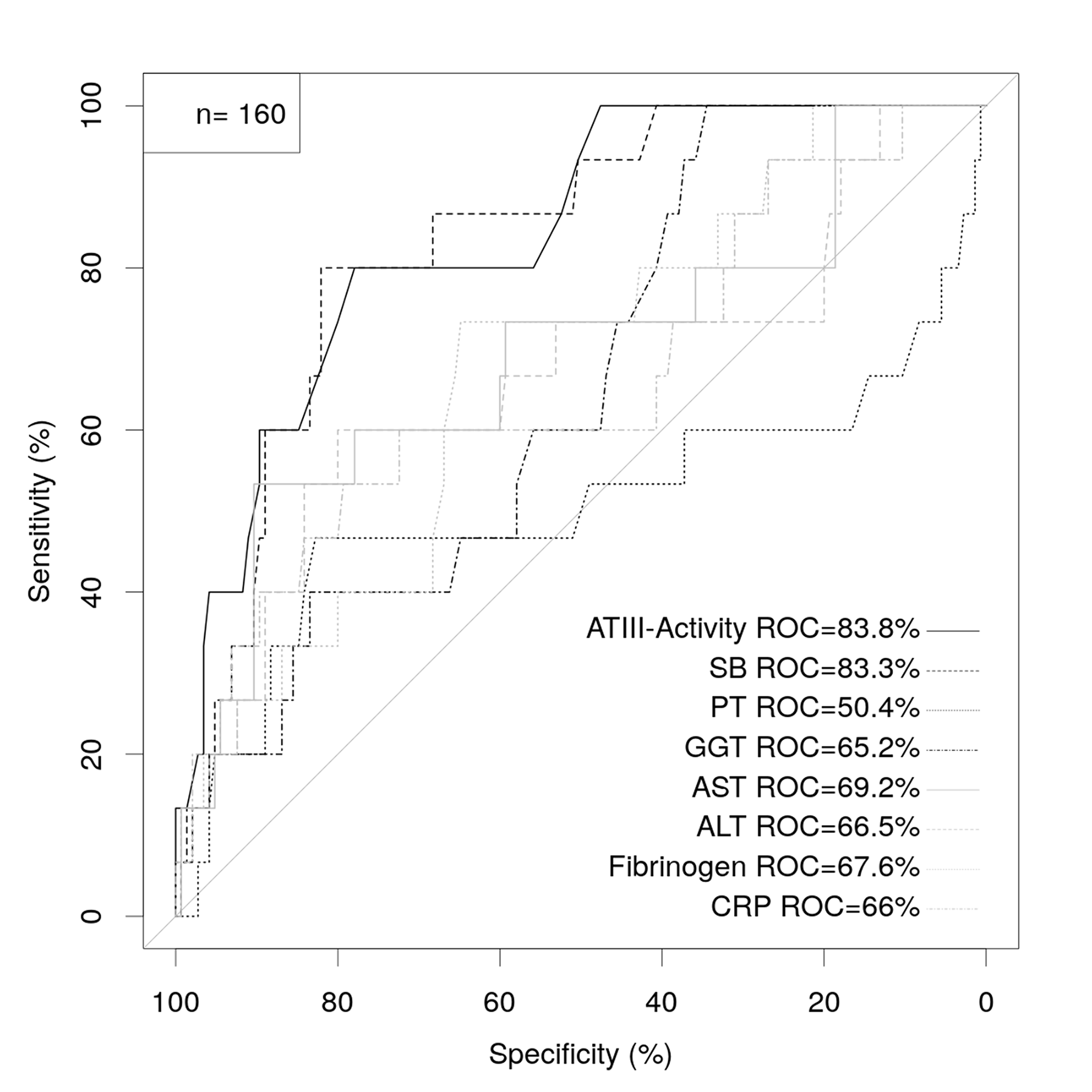

Supplement: S1 Fig — Predictive value of ATIII-activity, CRP, fibrinogen, SB, PT, AST, ALT and GGT on POD1 to predict postoperative LD using receiver operating characteristics (ROC) analysis was compared. (TIF) [file pone.0175359.s001.tif]

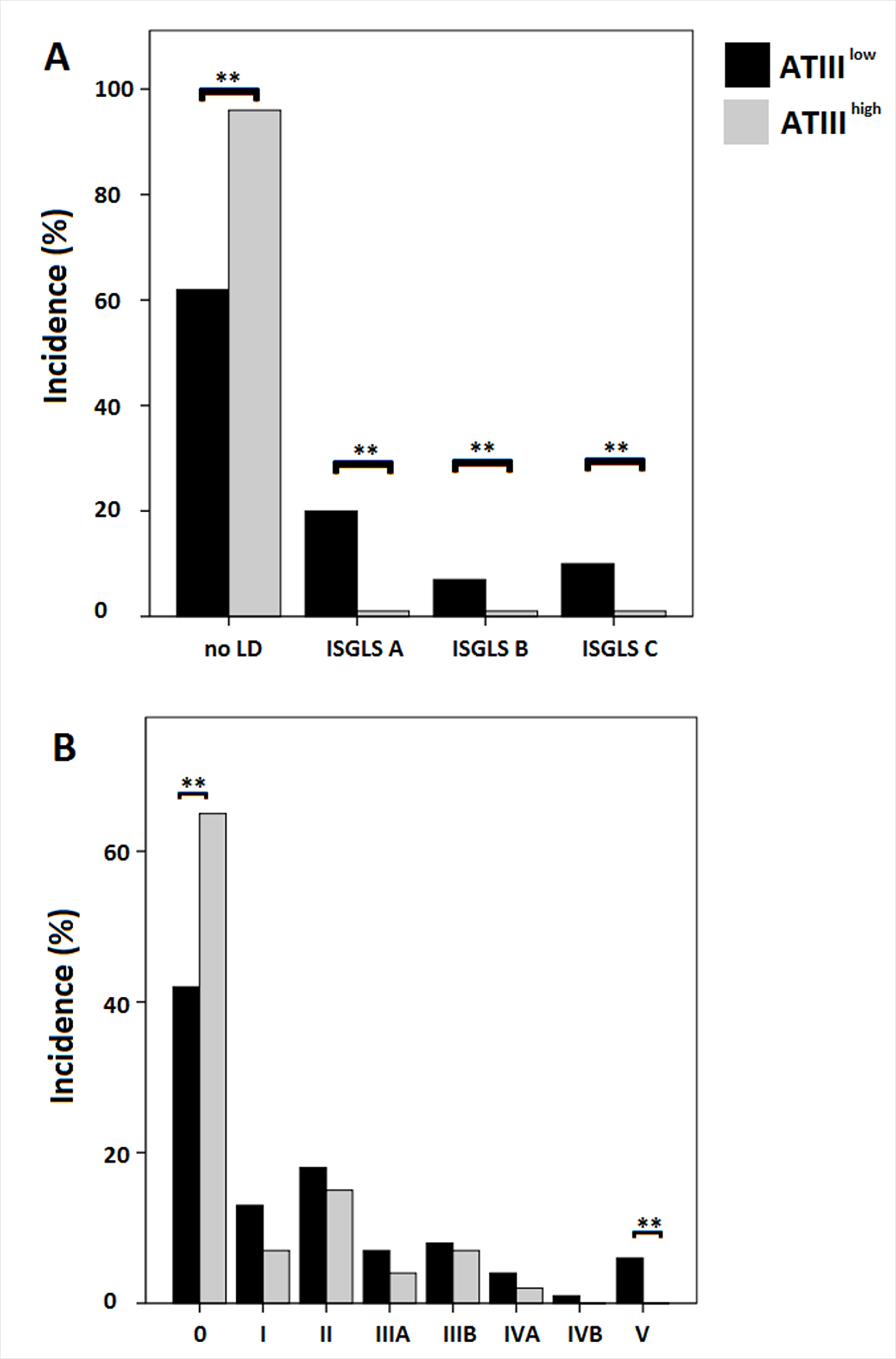

Supplement: S2 Fig — Patients were characterized in risk groups regarding their ATIII-activity levels, leading to a high-risk group (ATIIIlow) and a low-risk group (ATIIIhigh). Incidences of LD graded following the ISGLS score system were compared for patients in our high- and low-risk group (A). (no LD: P<0.001, 52 of 83 [62%] ATIIIlow vs 293 of 304 [96%] ATIIIhigh; ISGLS-A: P<0.001, 17 on 83 [20%] ATIIIlow vs 4 on 304 [1%] ATIIIhigh; ISGLS-B: P = 0.003, 6 on 83 [7%] ATIIIlow vs 4 on 304 [1%] ATIIIhigh; ISGLS-C: P<0.001, 8 on 83 [10%] ATIIIlow vs 3 on 304 [1%] ATIIIhigh). Incidences for morbidity grades according to Dindo et al. were compared to our risk groups. (no Morbidity: P<0.001, 35 on 83 [42%] ATIIIlow vs 196 of 304 [65%] ATIIIhigh; Dindo I: P = 0.063, 11 on 83 [13%] ATIIIlow vs 21 on 304 [7%] ATIIIhigh; Dindo II: P = 0.565, 15 on 83 [18%] ATIIIlow vs 47 on 304 [15%] ATIIIhigh; Dindo IIIA: P = 0.270, 6 on 83 [7%] ATIIIlow vs 13 on 304 [4%] ATIIIhigh; Dindo IIIB: P = 0.557, 7 on 83 [8%] ATIIIlow vs 20 on 304 [7%] ATIIIhigh; Dindo IVA: P = 0.504, 3 on 83 [4%] ATIIIlow vs 7 on 304 [2%] ATIIIhigh; Dindo IVB: P = 0.055, 1 on 83 [1%] ATIIIlow vs 0 on 304 [0%] ATIIIhigh; Dindo V: P<0.001, 5 on 83 [6%] ATIIIlow vs 0 on 304 [0%] ATIIIhigh). * P < 0.05; ** P < 0.005. (TIF) [file pone.0175359.s002.tif]
