# Supplementary material for: Early prediction of postoperative liver dysfunction and clinical outcome using antithrombin III-activity
Source: PLoS One. 2017 Apr 13;12(4):e0175359. doi: 10.1371/journal.pone.0175359 (PMC5391027; doi:10.1371/journal.pone.0175359)
Supplement: S1 Table — For further detail refer to PONE-D-16-33767R1_FTC-SupportingInformation.docx. (DOCX) [file pone.0175359.s003.docx]

**Supporting Information to:**

**Early Prediction of Postoperative Liver Dysfunction and Clinical Outcome Using Antithrombin III - Activity**

David Pereyra^1^, Florian Offensperger^1^, Florian Klinglmueller^2^, Stefanie Haegele^1^, Lukas Oehlberger^3^, Thomas Gruenberger^3^, Christine Brostjan^1^, Patrick Starlinger^1*^

^1^ Department of Surgery, Medical University of Vienna, General Hospital, Vienna, Austria

^2^ Core Unit for Medical Statistics and Informatics, Medical University of Vienna, Vienna, Austria

^3^ Department of Surgery I, Rudolfstiftung Hospital, Vienna, Austria

* corresponding author:

*Patrick Starlinger, MD, PhD*

*E-mail:* [*patrick.starlinger@meduniwien.ac.at*](mailto:patrick.starlinger@meduniwien.ac.at) *(PS)*

**Short Title:** Antithrombin III –Activity and Clinical Outcome after Liver Resection

**Keywords:** antithrombin III, postoperative liver dysfunction, liver regeneration, clinical outcome after liver resection.

**Table of contents**

S1 Table 3

| **S1 Table** | | | | | | | |
| --- | --- | --- | --- | --- | --- | --- | --- |
| **A. Missing data (evaluation cohort)** | | | | | | | |
| **LD** | **Time Point** | | **Mean**  **(ATIII, na.rm=T)** | **SD**  **(ATIII, na.rm=T)** | **NA** | **N** | **Missing Data** |
| No | PRE-OP | | 106.78022 | 15.93377 | 20 | 202 | 10% |
| No | POD 1 | | 74.76042 | 13.75496 | 10 | 202 | 5% |
| No | POD 5 | | 76.89773 | 16.54742 | 114 | 202 | 56% |
| Yes | PRE-OP | | 104.68000 | 14.43006 | 1 | 26 | 4% |
| Yes | POD 1 | | 54.84615 | 14.16670 | 0 | 26 | 0% |
| Yes | POD 5 | | 47.05000 | 16.14890 | 6 | 26 | 23% |
| **B. Missing data (validation cohort)** | | | | | | | |
| **LD** | **Time Point** | | **Mean**  **(ATIII, na.rm=T)** | **SD**  **(ATIII, na.rm=T)** | **NA** | **N** | **Missing Data** |
| No | PRE-OP | | 102.30935 | 14.58054 | 21 | 160 | 13% |
| No | POD 1 | | 72.43137 | 11.85758 | 7 | 160 | 4% |
| No | POD 5 | | 68.45679 | 18.32965 | 79 | 160 | 49% |
| Yes | PRE-OP | | 93.07692 | 19.65732 | 4 | 17 | 24% |
| Yes | POD 1 | | 56.37500 | 10.67005 | 1 | 17 | 6% |
| Yes | POD 5 | | 49.40000 | 27.29347 | 7 | 17 | 41% |
| **C. Missing data on outcome parameters (entire collective)** | | | | | | | |
| **PRE-OP** | | **Missing** | **N** | **LD** | **SM** | | **Mortality** |
|  |  | FALSE | 359 | 0.1058496 | 0.1476323 | | 0.02506964 |
|  |  | TRUE | 46 | 0.1086957 | 0.1956522 | | 0.02173913 |
| **POD1** | | **Missing** | **N** | **LD** | **SM** | | **Mortality** |
|  |  | FALSE | 387 | 0.10852713 | 0.1602067 | | 0.02583979 |
|  |  | TRUE | 18 | 0.05555556 | 0.0000000 | | 0.00000000 |
| **POD5** | | **Missing** | **N** | **LD** | **SM** | | **Mortality** |
|  |  | FALSE | 199 | 0.1507538 | 0.21105528 | | 0.040201005 |
|  |  | TRUE | 206 | 0.0631068 | 0.09708738 | | 0.009708738 |
| LD = liver dysfunction, ATIII = antithrombin III, SD = standard deviation, NA = not assessable, PRE-OP = preoperative, POD = postoperative day, SM = severe morbidity, POD = postoperative day. | | | | | | | |
